# Editorial: Challenges and current research status of vertigo/vestibular diseases, volume III

**DOI:** 10.3389/fneur.2025.1678805

**Published:** 2025-08-20

**Authors:** Linglin Zhou, Yujie Liu, Yunxiao Zheng, Jian Xin, Zixuan Yun, Shuhan Huang, Nicolas Perez-Fernandez, Andrea Castellucci, Hubertus Axer, Sulin Zhang, Jun Wang

**Affiliations:** ^1^Department of Otorhinolaryngology, Head and Neck Surgery, The First Affiliated Hospital, Jiangxi Medical College, Nanchang University, Nanchang, China; ^2^Department of Physiology, School of Basic Medicine, Huazhong University of Science and Technology, Wuhan, China; ^3^Department of Integrated Traditional Chinese and Western Medicine, Shaanxi University of Chinese Medicine, Shaanxi, China; ^4^Department of Otorhinolaryngology, Clinica Universidad de Navarra, Madrid, Spain; ^5^Department of Surgery, ENT Unit, Azienda USL-IRCCS, Reggio Emilia, Italy; ^6^Department of Neurology, Jena University Hospital, Jena, Germany; ^7^Department of Otorhinolaryngology, Union Hospital, Tongji Medical College, Huazhong University of Science and Technology, Wuhan, China

**Keywords:** vertigo, vestibular disorders, Benign Paroxysmal Positional Vertigo (BPPV), Meniere's disease (MD), Persistent Postural-perceptual Dizziness (PPPD), motion sickness (MS)

Vertigo/vestibular diseases comprise a spectrum of clinical syndromes spanning peripheral and central etiologies; these significantly increase fall risk and substantially compromise the quality of life of patients. This Research Topic collates 17 publications addressing the diagnosis, management, treatment, and pathophysiological mechanisms of prevalent vestibular pathologies, among others (Table 1).

**Table 1:** An overview of key information for 17 submitted articles.

**Author**	**Study type**	**Participants/materials**	**Methods**	**Main findings/objective**
Xing et al.	Prospective cohort study	HSC-BPPV patients (*n* = 553): 1. SRT (*n* = 110); 2. Head-shaking test + SRT (*n* = 112); 3. Bow and lean test + SRT (*n* = 114); 4. Seated supine positioning test + SRT (*n* = 108); 5. RART + SRT (*n* = 109)	1. The first test: alternative positional tests; 2. The second test: SRT	The RART outperformed other tests in eliciting nystagmus and identifying the affected semicircular canal in HSC-BPPV diagnosis, with an elicitation rate of 92.66% and accuracy of 85.45%.
Yang et al.	Computational mechanistic study combined with literature review	——	3-D computational modeling and literature review	In the Epley maneuver, sitting-up vertigo was often linked to treatment failure, while in the Semont maneuver, it was typically associated with successful treatment.
Álvarez De Linera-Alperi et al.	Retrospective longitudinal study	Unilateral MD patients (*n* = 137): 1. Cochleocentric group (*n* = 55); 2. Non-cochleocentric group (*n* = 82)	MRI hydrops assessments (3D-REALIR sequences)	Endolymphatic hydrops was more severe in the vestibule than in the cochlea in nearly 60% of the MD patients. Initial symptoms correlated with endolymphatic hydrops localization. Refined imaging could enhance the classification of cochleocentric and non-cochleocentric groups.
Yagi et al.	Cross-sectional observational study	1. PPPD coexisting with MD or other precipitants (*n* = 105): PPPD coexisting with MD (*n* = 23); 2. MD alone (*n* = 130)	1. Clinical symptom scales; 2. Vestibular function tests	PPPD patients experienced more severe subjective symptoms than MD-only patients, necessitating differentiation via clinical symptom scales. Vestibular function tests were normal in PPPD and mildly abnormal in MD.
Kim K. -T. et al.	Retrospective case series study	Patients with selective otolith dysfunction (*n* = 4)	Neurotologic evaluation; 4-h delayed 3D-FLAIR imaging	Selective otolith dysfunction accounted for ~5% of patients presenting with AVS. Diagnosis required extensive combined neurotologic evaluation and imaging.
Kim S. H. et al.	Retrospective cross-sectional observational study	1. Anterior inferior cerebellar artery infarction (*n* = 28); 2. Labyrinthitis (*n* = 51)	1. vHITs; 2. Other neurotologic evaluations; 3. Brain and inner ear MRI	Bilaterally positive vHITs were significantly associated with anterior inferior cerebellar artery infarction compared with labyrinthitis among patients with multiple vascular risk factors.
Kong et al.	Basic experimental research	1. Experimental: VGAT-IRES-Cre mice; 2. Control: wild-type mice	The RV-based retrograde tracing system	MVN GABAergic neurons received inputs from 60 brain nuclei, primarily in the cerebellum and medulla. MVN GABAergic neurons were regulated by the contralateral MVN, lateral vestibular nucleus, superior vestibular nucleus, and inferior vestibular nucleus.
Van Nechel et al.	Observational cohort study	1. BVD subjects (*n* = 38); 2. Control subjects free from vestibular or neurological disorders (*n* = 61)	Video Active Gaze Shift Test: a vHIT was utilized to record eye and head movements	Most BVD patients achieved efficient gaze stabilization during active gaze shifts by spontaneously reducing head speed to a stall speed and utilizing predictive compensatory mechanisms.
Shi et al.	Randomized controlled trial	College students with MS (*n* = 109): 1. Electric rotating chair group (*n* = 55); 2. Visual-motion cage rotating chair group (*n* = 54)	1. Electric rotating chairs training; 2. Visual-motion cage rotating chair training (90 s a day, 7 consecutive days)	Both methods relieved MS symptoms and enhanced sympathetic regulation. Electric-chair training yielded superior subjective relief, with greater benefit in low-susceptibility individuals, whereas high-susceptibility patients responded better to visual-motion cage training.
Gálvez-García et al.	Prospective experimental study	Healthy adults (*n* = 42)	1. No stimulation; 2. Sham GCS; 3. Sham tVNS; 4. Sham GCS and tVNS; 5. Active GCS condition; 6. Active tVNS; 7. Active GCS and tVNS	The combined application of tVNS and GCS was a more effective intervention method than either treatment alone, significantly alleviating the symptoms of SAS, improving body balance, and enhancing driving performance.
Wang et al.	Prospective experimental study	Participants were highly susceptible to VIMS (*n* = 46): 1. Experimental (*n* = 23); 2. Control (*n* = 20)	1. Experimental: VR-based cognitive ability training; 2. Control: activities unrelated to spatial cognition	VR training method effectively enhanced spatial cognitive abilities and alleviated VIMS symptoms.
Guo et al.	Cross-sectional study	1.Participants (5 age groups: 18–58 years, in 10-year increments) (*n* = 300); 2. A separate sample (*n* = 143)	1. Spatial ability tests: R-letter rotation test; 2. S-M mental rotation; 3. Surface development test; 4. Maze test	People's spatial ability generally declines with age, while women aged 28–37 performed better than other groups. Men's advantage over women varies across different tests of spatial ability. Different spatial tests assessed only partial abilities.
De Hertogh et al.	Perspective review	——	——	An overview of the pathophysiology, diagnostic challenges, and therapeutic strategies for CGD.
Hou et al.	Study protocol	Participants diagnosed with cervical vertigo with qi-blood deficiency	1. TNRM-UN (*n* = 33); 2. Three-needle regulating the mind combined with traditional acupuncture group (*n* = 33); 3. Umbilical needle group (*n* = 33)	To assess the safety and effectiveness of the TNRM-UN therapy for cervical vertigo with qi-blood deficiency, and to determine whether it is superior to current acupuncture treatments.
Liang et al.	Cross-sectional study	Participants from NHANES (1999-2004) (*n* = 6970)	1. Weighted logistic regression; 2. trend tests; 3. Restricted cubic spline analysis; 4. Subgroup analysis	Elevated homocysteine, hyperhomocysteinemia and H-type hypertension were significantly associated with various symptomatic dizziness.
Situkho et al.	Systematic review protocol	Studies on humans of any age on PLF containing data with diagnostic test accuracy estimation or in which diagnostic test accuracy could be calculated.	1. Eligibility criteria; 2. Search strategy; 3. Data extraction; 4. Assessment of the risk of bias; 5. Data synthesis; 6. Assessment of confidence in cumulative evidence	Provide comprehensive evidence on diagnostic test accuracy for PLF.
Strupp et al.	Observational cross-sectional study	Replies of questionnaire (*n* = 234; five continents, 47 countries, 162 cities, and 188 centers)	A web-based standardized survey questionnaire of six common peripheral vestibular disorders (BPPV, AUVP, MD, BVP, VP, SCDS)	There was significant heterogeneity in the treatment of six common peripheral vestibular disorders. Evidence gaps were large, and well-designed controlled trials were needed.

HSC-BPPV, horizontal semicircular canal benign paroxysmal positional vertigo; SRT, supine roll test; RART, rapid axial roll test; MD, Meniere's disease; PPPD, persistent postural-perceptual dizziness; AVS, acute vestibular syndrome; vHIT, video head impulse test; VR, virtual reality; MVN, medial vestibular nucleus; BVD, bilateral vestibular deficiency; MS, motion sickness; GCS, Galvanic Cutaneous Stimulation; tVNS, Transcutaneous Vagal Nerve Stimulation; SAS, Simulator Adaptation Syndrome; VIMS, visually induced motion sickness; CGD, cervicogenic dizziness; TNRM-UN, three-needle of regulating the mind combined with umbilical needle; PLF, Perilymphatic Fistula; AUVP, acute unilateral vestibulopathy; BVP, bilateral vestibulopathy; VP, vestibular paroxysmia; SCDS, superior canal dehiscence syndrome.

This field encompasses two studies on Benign Paroxysmal Positional Vertigo (BPPV). Xing et al. conducted a comparative analysis between the supine roll test (SRT) and alternative positional tests for diagnosing horizontal semicircular canal BPPV (HSC-BPPV). Their analysis identified the reverse autorotation test (RART) as the most accurate tool, demonstrating significant advantages in nystagmus elicitation sensitivity and affected canal determination accuracy. Consequently, they recommend RART as the preferred alternative to SRT in clinical practice. The Tumarkin-like phenomenon refers to a transient vestibular crisis characterized by sudden dizziness, postural instability, and sensations of falling, which may occur during the final step of repositioning maneuvers for BPPV (1). Yang et al. explored the mechanisms and clinical significance of this phenomenon during the Epley and Semont maneuvers. Using virtual simulations and a literature review, their results indicated that in the Epley maneuver, the Tumarkin-like phenomenon frequently correlated with treatment failure, whereas in the Semont maneuver, it was typically associated with successful treatment.

There are two studies focusing primarily on Ménière's disease (MD). Álvarez De Linera-Alperi et al. found that endolymphatic hydrops was more severe in the vestibule than the cochlea in nearly 60% of cases, correlating with initial symptoms, but no significant differences in auditory or vestibular function tests were found during follow-up. This challenges the traditional cochleocentric progression theory and suggests a more complex pathophysiology of MD requiring further investigation. Persistent Postural-Perceptual Dizziness (PPPD) is a chronic functional vestibular disorder that commonly manifests secondary to acute or episodic vestibular syndromes. Yagi et al. demonstrated that patients with comorbid MD and PPPD exhibited more severe subjective symptoms relative to isolated MD. This evidence underscored the necessity of differentiating these conditions using clinical symptom scales, thereby enabling targeted therapeutic strategies specific to PPPD.

This field includes two studies investigating acute vestibular syndrome (AVS). Kim K.-T. et al. reported four cases of patients with selective otolith dysfunction presenting with acute spontaneous vertigo, a condition accounting for approximately 5% of AVS presentations yet easily overlooked. Comprehensive neurotologic evaluation combined with dedicated inner ear MRI facilitated the detection of selective otolithic dysfunction, expanding the clinical spectrum of AVS. Furthermore, acute audiovestibular syndrome (AAVS) is characterized by acute vertigo, tinnitus, aural fullness, or hearing loss persisting over 24 h, which may result from benign inner ear disorders or posterior circulation strokes (2). Early differentiation is critical to minimize neurological sequelae and preserve auditory function. Kim S. H. et al. demonstrated that patients with anterior inferior cerebellar artery infarction exhibited bilateral positive video head impulse test (vHIT) more frequently than those with labyrinthitis, particularly among individuals with multiple vascular risk factors.

Furthermore, two additional studies pertain to compensatory mechanisms of vestibular disorders. GABAergic neurons within the medial vestibular nucleus (MVN) contribute to the rebalancing of the commissural system, thereby alleviating acute peripheral vestibular dysfunction syndrome (3, 4). However, the specific neural circuits providing synaptic input to these neurons remain unclear. Kong et al. identified 60 nuclei projecting to MVN GABAergic neurons in mice, primarily localized in the cerebellum and the medulla. Additionally, MVN GABAergic neurons were regulated by the contralateral MVN, lateral vestibular nucleus, superior vestibular nucleus, and inferior vestibular nucleus. These findings advance the understanding of vestibular dysfunction at the neural circuit level and may promote vestibular compensation strategies. Van Nechel et al. found that patients with bilateral vestibular deficiency (BVD) achieve functional gaze stability during active head movements by reducing head speed and utilizing predictive eye movements. The video Active Gaze Shift Test revealed compensatory mechanisms invisible to passive tests, offering insight into compensatory eye movements and symptom understanding.

Additionally, four articles focus on motion sickness (MS) and virtual reality (VR) in this topic. MS arises from vestibular dysfunction induced by sensory conflict between perceived and actual motion, manifesting as a constellation of autonomic and vestibular symptoms, including nausea, dizziness, vomiting, cephalalgia, and fatigue (5). Shi et al. demonstrated that two vestibular function training methods significantly alleviated MS symptoms. The electric rotating chair was superior to the visual-motion cage rotating chair in improving subjective discomfort. Subgroup analysis revealed that for low-susceptibility individuals, the electric rotating chair was more effective, while for high-susceptibility individuals, the visual motion cage rotating chair showed better improvement, indicating the need for therapeutic individualization. Gálvez-García et al. reported that the combined application of transcutaneous vagus nerve stimulation (tVNS) and galvanic cutaneous stimulation (GCS) reduced simulator adaptation syndrome (SAS) symptoms more effectively than either intervention alone, suggesting a novel strategy for SAS management. Visually induced motion sickness (VIMS) has increased following the widespread adoption of VR. Wang et al. found that VR-based spatial cognition training significantly improved spatial abilities and reduced VIMS symptoms in the experimental group, with no improvement observed in controls. Guo et al. investigated the impact of age on spatial abilities across genders using VR technology, providing insights for occupational preference stratification.

Moreover, within this topic, three articles center on dizziness. Cervicogenic dizziness (CGD), a debated clinical entity characterized by dizziness associated with cervical pain or dysfunction, arises from altered proprioceptive input originating in the cervical spine (6). De Hertogh et al. conducted a perspective review of CGD, covering its pathophysiology, diagnostic challenges, and therapeutic strategies. In Traditional Chinese Medicine, qi-blood deficiency is the dominant pattern of CGD, resulting in brain-marrow malnourishment and often accompanied by anxiety or depression. To rigorously evaluate a potential therapeutic intervention, Hou et al. conducted a research protocol aiming to determine the efficacy and safety of three-needle regulating the mind combined with umbilical needle (TNRM-UN) therapy for CGD patients with qi-blood deficiency. What is more, Liang et al. demonstrated significant associations between homocysteine levels, hyperhomocysteinemia, and H-type hypertension with various symptomatic dizziness. Consequently, early detection and management of hyperhomocysteinemia and H-type hypertension are crucial for dizziness management and diagnosis.

Perilymphatic Fistula (PLF) is an inner ear disorder involving abnormal leakage of perilymph into the middle ear. Currently, the diagnosis of PLF lacks internationally established criteria and relies on clinical assessment and functional testing. To address this gap, Situkho et al. developed a research protocol designed to systematically evaluate the accuracy of diagnostic tests for PLF. Complementarily, Strupp et al. performed a standardized survey across 188 international centers, revealing substantial heterogeneity in managing six peripheral vestibular disorders. These findings underscore substantial evidence gaps and highlight the urgent need for standardized therapeutic protocols supported by well-designed controlled trials to establish evidence-based care.

In summary, this Research Topic assembles a series of articles on vertigo or vestibular diseases, delineating recent advances in the field to pave the way for the development of novel therapeutic and management strategies for these disorders.
